# Educational outcomes of children in contact with social care in England: a systematic review

**DOI:** 10.1186/s13643-019-1071-z

**Published:** 2019-06-28

**Authors:** Matthew A. Jay, Louise Mc Grath-Lone

**Affiliations:** 1 UCL Legal Epidemiology Group, UCL Great Ormond Street Institute of Child Health, 30 Guilford Street, London, WC1N 1EH UK; 2 Department of Anaesthesia and Pain Medicine, Great Ormond Street Hospital for Children NHS Foundation Trust, Great Ormond Street, London, WC1N 3JH UK; 3 Rees Centre, University of Oxford, 15 Norham Gardens, Oxford, OX2 6PY UK

**Keywords:** Social care, Children in need, Looked after children, Children Act, Education, School

## Abstract

**Background:**

In England, the state intervenes in the lives of children through Children’s Social Care (CSC) services with the aim of supporting and maintaining their welfare. It is known from government cross-sectional data that children who experience these CSC interventions (such as state care) have consistently poorer educational outcomes than the general population. However, these data are limited in providing crude estimates of association and in ignoring longitudinal histories. This systematic review aimed to appraise the extant research evidence from longitudinal studies and answer the question: how do educational outcomes differ between children in contact with CSC and the general population in the UK?

**Methods:**

According to a pre-defined protocol, we searched 16 health, social care, education and legal databases for population-level quantitative studies conducted on UK children with exposure to CSC, a general population comparison group and an educational outcome. We also conducted snowball searches and searches of Google Scholar and grey literature. Data on whether each study met inclusion criteria were extracted, and findings of included studies were synthesised narratively. Risk of bias was assessed using the National Institutes of Health Quality Assessment Tool for Observational Cohort and Cross-Sectional Studies.

**Results:**

In total, 5482 sources were screened which resulted in seven studies being included in the narrative synthesis. Only three were published in peer-reviewed journals. All but one used administrative education data and five used administrative data from CSC services. In all studies, exposure to CSC interventions was measured crudely, ignoring heterogeneity in the experiences of children. All agreed that children in contact with CSC services perform worse than their peers on all outcomes (variously: exam results, absences, exclusions, school moves, being missing from school, higher education aspirations and quality of school).

**Conclusions:**

Despite employing a search across 16 databases supplemented with additional searches of other online sources, we found only seven studies that met our inclusion criteria. This review throws into sharp relief the urgent need to conduct more population-level research into the educational prospects of children in contact with CSC services.

**Systematic review registration:**

PROSPERO CRD42018089755

**Electronic supplementary material:**

The online version of this article (10.1186/s13643-019-1071-z) contains supplementary material, which is available to authorized users.

## Background

In England, the state intervenes in the lives of children through Children’s Social Care (CSC) services with the aim of supporting and maintaining their welfare. The principal legislation by which this is achieved is the Children Act 1989 which vests in local authorities a range of powers and duties in respect of children ‘in need’ (CiN) and children who are ‘looked after’ (CLA). A CiN is essentially any child who is disabled or unlikely to achieve or maintain a reasonable standard of health or development (or whose health or development is likely to be impaired) without the provision of local authority services. This can include children subject to child protection plans because they are thought to be at risk of harm. A CLA is a child whom the local authority accommodates and/or for whom it cares. Some children can be looked after where the parents agree (or there are no parents) so long as to do so would safeguard or promote the child’s welfare. Others are looked after because a court has issued a care order, enabling the local authority to care for and accommodate the child without the need for parental consent. In the latter case, it is a requirement that the child be suffering, or likely to suffer, significant harm due to the care of the parents. Whether a child meets the statutory criteria for support under any of these provisions is determined by local authority social workers following referral. Local authorities are involved with families for a range of different reasons such as poverty, disability or illness and abuse or neglect.

Annual statistical releases from the Department for Education (DfE) show that these two main groups of children involved with CSC services have consistently poorer educational outcomes than the general population [1], and given that educational success is key to a range of health and other outcomes [2] this is particularly concerning. The most recent available statistics show that in 2017, just 2.7% of children who had been looked after for 12 months or more and 3.9% of CiN achieved the English Baccalaureate (i.e. General Certificates of Secondary Education [GCSEs] in English language and literature, maths, science, geography or history, and a language; GCSEs are sat at age 15/16 at the end of secondary schooling) compared to 21.9% of the general population. Children who have been looked after for 12 months or more are, however, comparable to the general population in being classified as persistent absentees (about 10% each in 2017) whereas CiN are much more likely to be so (30% in 2017) [3].

The purpose of the annual DfE statistics is to monitor the provision of services and assess the effects of policies designed to improve educational outcomes for CiN and CLA, such as exam results, absenteeism and exclusions and quality of school [4]. However, their utility in terms of evaluating policy and improving outcomes is limited by the scope of children that are included. Firstly, DfE statistics are cross-sectional and so only include children who are looked after or in need on a particular date in time, namely on 31 March of each year. This lack of longitudinal perspective means current evidence about the educational outcomes of CiN and CLA does not account for the time-varying nature of these forms of involvement with CSC services. A further limitation of these high-level statistics is that they ignore differences between sub-groups of CiN and CLA and do not take into account how variation in their longitudinal ‘careers’ of contact with CSC services might be related to their educational outcomes. For example, a systematic review by O’Higgins et al. [5] examining the factors associated with educational outcomes of children in foster care found that lower attainment was associated with a longer duration in care. DfE statistics also exclude the numerous vulnerable children who are in contact with CSC services but do not meet the thresholds for being designated in need or becoming looked after. For example, in 2017, 646,120 children were referred to CSC but 38.0% were not designated as a CiN or CLA that year (DfE CiN statistical release, Main Tables, Table A1, Cell J29-J37 [6]). Comparing different sub-groups of children based on relevant factors such as the nature of their trajectories over time is necessary in order to better direct interventions for improving outcomes. To date, however, there has been no systematic evaluation of the evidence base of the educational outcomes of all children in contact with CSC services.

## Methods

### Aims

Given the limitations of DfE’s annual statistics, this systematic review aims to review the extant research evidence from longitudinal studies of the relationship between involvement with CSC services and educational outcomes. This systematic review had one broad question: how do educational outcomes differ between children in contact with CSC services and the general population in the United Kingdom (UK)?

### Protocol and registration

Prior to commencing this review, we developed a protocol which was registered with the PROSPERO international prospective register of systematic reviews (registration number CRD42018089755) [7]. This review has been reported in accordance with the PRISMA statement (Additional file 1) [8]. No amendments were made to the protocol after its registration other than that we carried out an updated search (details below).

### Eligibility criteria

Full eligibility criteria and rationale are given in Table 1. In brief, we included population-level quantitative studies conducted on UK children with exposure to CSC, a general population comparison group and an educational outcome. Only UK studies were included as differences in law, social policy and the underlying populations render cross-country comparisons difficult.

**Table 1:** Eligibility criteria and rationale

Criterion	Rationale
1. Is a primary quantitative research study.	Although qualitative work can provide important insights into how systems operate, only quantitative studies provide estimates of interest at a population level. Mixed-methods studies were eligible if the quantitative component met the eligibility criteria. ‘Primary’ research was any research that used de novo data collection or analysis of record-level administrative data.
2. Has an educational outcome (i.e. attainment, exclusion, absenteeism, quality or type of school or participation in education beyond compulsory age).	This broad range of eligible educational outcomes was selected to include all facets of educational experience and success that are considered important for children in contact with CSC in current UK policy, as evidenced by the Department for Education statutory guidance on promoting the education of looked-after children and those previously looked-after. [9]
3. Main exposure is referral to CSC (any contact, including, e.g., ‘child in need’ assessments and provision, child protection investigations and care).	Exposure to CSC was also defined broadly as we made no a priori assumptions as to how studies would measure this.
4. Has a concurrent comparison group of the general population/children with no contact with CSC.	Uncontrolled studies do not provide an estimate of associations.
5. Sample or population studied is age < 18.	This study only examined childhood education.
6. Any study design.	We include all study designs, subject to the above criteria (e.g. cohort and cross-sectional designs); we did not expect any randomised-controlled trials.
7. English-language only.	Limited by the investigators’ languages. Only studies on UK populations were included (criterion 8) so this is unlikely to have been a biasing factor.
8. Conducted in any UK population.	Differences in social policy render cross-country comparisons difficult and international studies provide limited information regarding outcomes of children in the UK.
9. Conducted in 1991 onwards.	The law regarding the welfare of children in England was overhauled by the Children Act 1989, which came almost fully into force on 14 October 1991 (similar provisions were latterly enacted in the other three UK countries).
10. In a peer-reviewed source or not.	We were aware a priori that some studies in this area are published as non-peer-review reports. We did not therefore limit our search to peer-reviewed sources only.

*CSC* children’s social care

### Information sources and search strategy

On 21 December 2017, MAJ searched 16 health, social care, education and legal databases, the names and date coverage of which are given in Table 2. Full search strings are available in Additional file 2. We adapted a search strategy developed by O’Higgins et al. [5] and applied it across all databases. References were imported into the Mendeley reference management software with the exception of the results from Westlaw where this was not possible (these abstracts were downloaded into a word document for screening). We also carried out a ‘snowball’ search [10] by searching the reference lists of the full texts for additional studies and using Google Scholar to identify and screen studies citing them.

**Table 2:** Databases searched

Database	Coverage
Ovid	
Medline and Epub Ahead of Print, In-Process & Other Non-Index Citations, Daily and Versions	1946 to present
Embase and Embase Classic	1947 to present
PsycInfo	1806 to present
Social Policy & Practice	1890s to present
Scopus	1788 to present
EBSCOhost	
British Education Index	1929 to present
Education Abstracts	1983 to present 1995 to present (books)
The Education Resources Information Center	1966 to present
Index to Legal Periodicals and Books	1979 to present
ProQuest Central	
The Education Database	1988 to present
Social Science Database	1942 to present
The Applied Social Sciences Index & Abstracts	1987 to present
The International Bibliography of the Social Sciences	1951 to present
The Sociology Database	1985 to present
Sociological Abstracts	1952 to present
Westlaw UK	1986 to present

Note that coverage years are given for information only: records from before 1991 were excluded as per the study protocol

Because we were already aware of some publications that were not in peer-reviewed sources, we also carried out additional searches for grey literature. On 26 April 2018, we conducted a search of Google Scholar and additional supplementary searches for publications on websites of ten relevant organisations (including government departments, charities, think-tanks and research institutes). Full details of these supplementary searches can be found in the Additional file 2. Finally, we updated the database search on 7 May 2019 and the snowball and additional searches on 10 May 2019 as detailed in Additional file 3. We used the same search method, except that we narrowed the searches to 2017 onwards.

### Study selection, data collection, risk of bias assessment and data items

All identified titles and abstracts were independently screened by the two authors (MAJ and LMcGL) using a data collection tool created in Google Forms. This tool, which was piloted on the first 50 abstracts, had checkboxes to assess each record against the eligibility criteria as well as space to write brief comments. Each reviewer screened 100% of the records independently. Screening results were exported into Microsoft Excel and disputes were resolved by discussion; there was no need for disputes to be referred to a third reviewer. Full texts of potentially eligible studies were downloaded and all were read by MAJ and LMcGL independently and results were synthesised narratively through discussion.

Studies deemed eligible were then assessed for risk of bias using the National Institutes of Health Quality Assessment Tool for Observational Cohort and Cross-Sectional Studies [11]; this was done for all included studies by MAJ and LMcGL independently. This tool assesses studies against a range of criteria including, inter alia, definition of the target population, response rates, validity and reliability of measures, loss to follow up and controlling of confounders; it also asks for an overall assessment as either good, fair or poor. MAJ and LMcGL reached consensus after discussion and referral to a third reviewer was not required.

### Summary measures, synthesis and risk of bias across studies

Due to the broad nature of our search strategy, we did not specify a priori any particular summary measures (e.g. risk ratios) to be extracted. We intended to synthesise results, including risk of bias across studies, narratively. To do so, both authors read all included studies in full and discussed between them the strengths and limitations of each, including with reference to their risk of bias. Narrative synthesis was chosen because we expected heterogeneity in, for example, outcomes chosen. Additionally, after screening was complete, there were too few studies to conduct meta-analysis. Due to the number of studies eventually included in the synthesis, we did not explicitly group studies, though similarities and differences in study design are commented upon where appropriate.

## Results

### Study selection

**Fig. 1:**
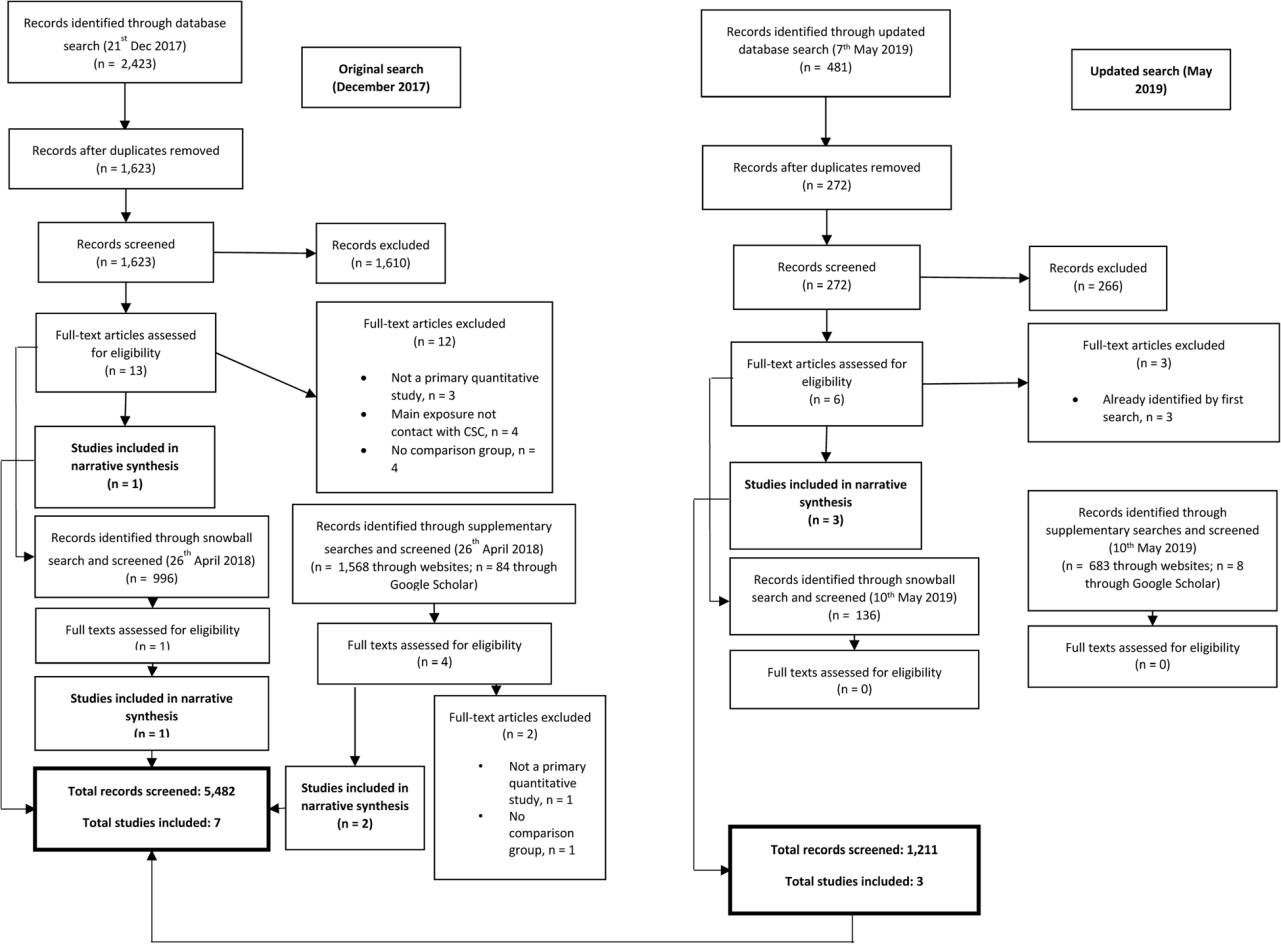
Flow diagram of studies screened and included

In the first main search, 2423 studies were identified in the database search (a flow diagram is given in Fig. 1). After deduplication, 1623 records were screened and 1610 of these were excluded. Thirteen full texts were assessed for eligibility and 12 were excluded: three were not primary quantitative studies, contact with CSC was not the main exposure in four, four had no comparison group, and one was conducted on a non-UK population. These references and reasons for exclusion are given in Additional file 2. For the 13 potential full texts, we screened publications included in their reference lists (*n* = 461) and that cited them as references (*n* = 535); from this snowball search, we identified one further eligible publication [12].

Our supplementary searches of Google Scholar and other relevant websites resulted in 1652 sources being identified. Following screening, nine additional sources were identified on the Department for Education website; however, as eight of these were annual reports of cross-sectional statistics, we collated these into one source for the purposes of narrative synthesis.

The updated searches in May 2019 resulted in a total for 1211 records being screened and three new, eligible studies being identified; these searches are shown in in Fig. 1, with full details in Additional file 3. In total, therefore, we screened 5482 records which resulted in seven studies being included in the narrative synthesis [12–18].

### Study characteristics

The studies are summarised in Table 3. One consists of the series of eight statistical releases by the DfE [14], one is a research report of longitudinal data by the DfE [13], two were reports self-published by a research centre [12] and a children’s charity [16] and three were peer-reviewed journal articles [15, 17, 18]. The DfE statistical releases are annual cross-sectional population-level snapshots of Key Stage results and exclusion and absence rates.

**Table 3:**
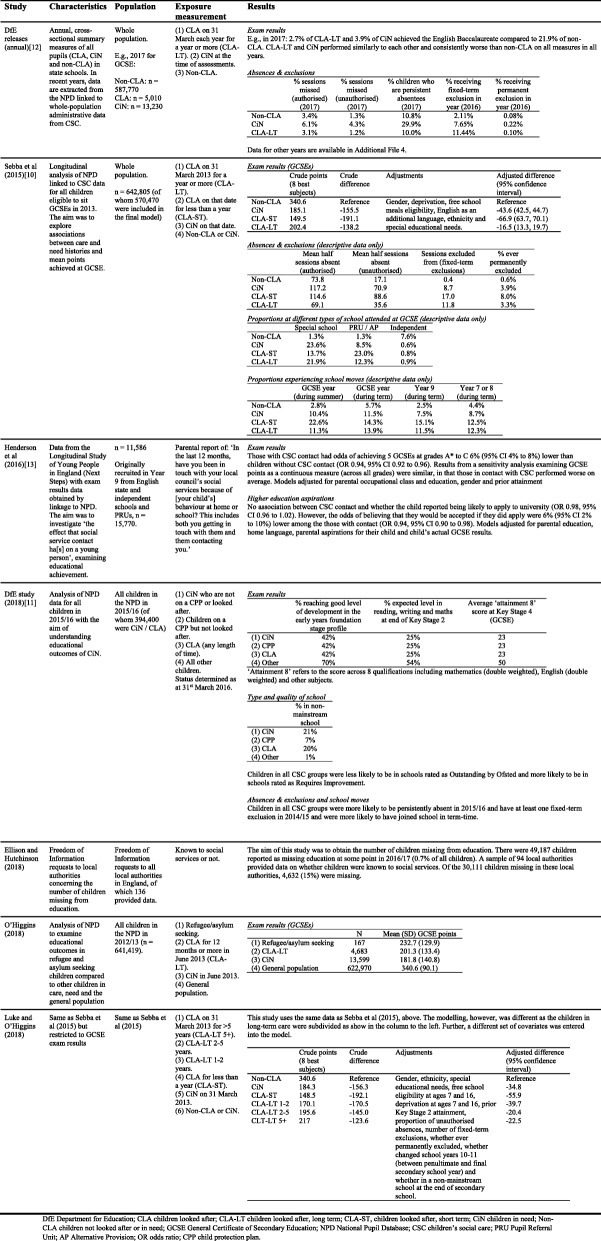
Summary of studies

### Risk of bias assessment

Results from the risk of bias assessment of the four studies are given in Table 4. All had clearly stated research questions and ‘recruited’ participants from clearly specified and defined populations with high response rates. In the cases of the DfE statistical releases [14], DfE research report [13], Sebba et al. [12], O’Higgins [18] and Luke and O’Higgins [17], data were available on all or almost all children in the target population. In Henderson et al. [15], which used the Next Steps study, the response rate at wave 1 was 74% [19]. Ellison and Hutchinson [16] employed freedom of information requests to all English local authorities with a response rate of 62% for the component of their study involving CSC data. None provided an explicit sample size justification as they used all data available. However, this does not detract from study quality, except perhaps in the case of Ellison and Hutchinson [16] where this is a higher risk of non-response bias, as the sample sizes were sufficiently large to support the analyses undertaken, and in the case of Sebba et al. [12], Luke and O’Higgins [17] and Henderson et al. [15], point estimates with standard errors are presented.

**Table 4:** Quality assessment of included studies

Criterion	DfE statistical releases (ann.) [13]	Sebba et al. [11]	Henderson et al. [14]	DfE study [12]	Ellison and Hutchinson [16]	O’Higgins [17]	Luke and O’Higgins [18]
1. Was the study research question or objective in this paper clearly stated?	Yes	Yes	Yes	Yes	Yes	Yes	Yes
2. Was the study population clearly specified and defined?	Yes	Yes	Yes	Yes	Yes	Yes	Yes
3. Was the participation rate of eligible persons at least 50%?	Yes	Yes	Yes	Yes	Yes	Yes	Yes
4. Were all the subjects selected or recruited from the same or similar populations (including the same time period)? Were inclusion and exclusion criteria for being in the study prespecified and applied uniformly to all participants?	Yes	Yes	Yes	Yes	Yes	Yes	Yes
5. Was a sample size justification, power description, or variance and effect estimates provided?	No	Yes	Yes	No	NA	No	Yes
6. For the analyses in this paper, were the exposure(s) of interest measured prior to the outcome(s) being measured?	Yes	Yes	Yes	Yes	CD	Yes	Yes
7. Was the timeframe sufficient so that one could reasonably expect to see an association between exposure and outcome if it existed?	CD	CD	CD	CD	NA	CD	CD
8. For exposures that can vary in amount or level, did the study examine different levels of the exposure as related to the outcome (e.g. categories of exposure, or exposure measured as a continuous variable)?	No	Yes	No	No	No	No	Yes
9. Were the exposure measures (independent variables) clearly defined, valid, reliable and implemented consistently across all study participants?	Yes	Yes	CD	Yes	No	Yes	Yes
10. Was the exposure(s) assessed more than once over time?	No	No	Yes	No	CD	No	No
11. Were the outcome measures (dependent variables) clearly defined, valid, reliable and implemented consistently across all study participants?	Yes	Yes	Yes	Yes	CD	Yes	Yes
12. Were the outcome assessors blinded to the exposure status of participants?	Yes	Yes	Yes	Yes	No	Yes	Yes
13. Was loss to follow-up after baseline 20% or less?	NA	Yes	No	Yes	NA	Yes	Yes
14. Were key potential confounding variables measured and adjusted statistically for their impact on the relationship between exposure(s) and outcome(s)?	No	Yes	Yes	No	No	No	Yes
Overall assessment	Fair	Good	Fair	Fair	Poor	Fair	Good

*CD* cannot determine, *NA* not applicable

One key difference between the longitudinal studies was in loss to follow up rates: in Henderson et al. [15], 27% of children had been lost by wave 4. Statistical weighting was employed but there may still be bias unaccounted for. By contrast, Sebba et al. [12] had no loss to follow up as they used whole-population data. However, children were ‘followed up’ retrospectively and only children with complete data (including at Key Stage 2 exams (age 11) and at GCSE (age 16)) were included in the models (*n* = 570,470 (89%) children). Luke and O’Higgins [17] and O’Higgins [18] used essentially the same data. There is therefore a potential selection bias if CLA or CiN are less likely to sit their exams, which is likely given the higher rates of exclusions and SEN among the looked after and in need groups. The DfE reports [13, 14] and Ellison and Hutchinson [16] are each cross-sectional and therefore not subject to loss to follow up as such.

In all, exposure status was measured prior to the outcome (though in different ways) except Ellison and Hutchinson [16], where this could not be determined from the report. The DfE releases [14] compare children who are looked after on 31 March each year and have been for 12 months or more. For some recent years, children who were in need at the time of outcome ascertainment (and therefore were assessed as being in need prior to this) were also examined. However, these are blunt definitions that do not allow examination of any kind of ‘dose-response’ relationship and they do not include children who are looked after for less than 12 months or children who were looked after but not on 31 March. The recent DfE report [13] also focused on children who were looked after or designated in need on 31 March 2016; however, it includes all CiN and CLA regardless of the length of time they had been involved with CSC. The group of CiN was also further disaggregated into two groups: those who were subject to a Child Protection Plan and those who were not. Sebba et al. [12] used the same data source as the DfE statistical releases and report but provided a more nuanced classification, viz. (1) CLA on 31 March 2013 for a year or more, (2) CLA on that date for less than a year, (3) CiN on that date, or (4) not in need or care on that date. Although this classification does allow for analysis by different levels of exposure, it still does not represent the full range of naturally occurring sub-groups of care and need trajectories which may be related with educational outcomes. Luke and O’Higgins [17], who, as previously outlined, used the same data as Sebba et al. [12], also used the same exposure categorisation except they further divided the long-term group into subgroups. The focus of O’Higgins [18] was asylum-seeking or refugee children in care for a year or more, compared to other CLA in care for a year or more, CiN and the general population. Exposure ascertainment for all of these sources was from administrative data from CSC recorded in ‘real time’ and therefore limits the risk of misclassification bias.

In Ellison and Hutchinson [16], the exposure is defined as whether a child is known to social services; however, it is not clear from the report how this was defined or ascertained by the local authority respondents. In Henderson et al. [15] exposure status was ascertained at waves 2 and 3 (GCSE results being measured between waves 3 and 4) by asking the main parent whether, in the previous 12 months, they had been in touch with their local council’s social services because of their child’s behaviour at home or school including where the parent or the council initiated contact. It is not clear whether any contact exclusively relates to social work intervention or what interventions, if any, were received. The question implicitly excluded contact with CSC services for reasons other than behaviour. As a self-reported measure of a sensitive subject, this question might also be subject to recall and desirability bias. This is therefore a crude indicator of CSC intervention, which was the study’s main objective, and one without data presented as to its validity or reliability.

All studies except Ellison and Hutchinson [16] examined attainment, which they ascertained in the same way using GCSE data from the National Pupil Database [20]. GCSEs are marked by independent examiners who are blind to the identity of the pupil. The DfE data [13, 14] and Sebba et al. [12] also used the National Pupil Database to ascertain absences and exclusions. Sebba et al. [12], Luke and O’Higgins [17] and Henderson et al. [15] employed multivariable analysis methods. In the cases of Sebba et al. [12] and Luke and O’Higgins [17], a range of child- and school-level confounders, measured in a non-time-varying way, were adjusted for including child demographics, prior attainment, special educational needs status, school type, free school meal eligibility and area-based deprivation. Henderson et al.’s [15] models adjusted for parental socioeconomic position, gender and prior attainment. The other studies presented descriptively and without any confounder adjustment.

### Outcomes of included studies

Table 3 provides the results from each study (in the case of the DfE releases [14], data for the latest available year are presented in Table 3 and data for all years can be found in Additional file 4). All studies agree that children with contact with CSC, as respectively defined, perform worse than their peers on all outcomes (variously: exam results, absences, exclusions, school moves, being missing from school, higher education aspirations and quality of school). Where differences between CLA and CiN were examined, CiN had similar outcomes to CLA and in some cases worse.

## Discussion

Despite employing a search across 16 databases supplemented with additional searches of other online sources, we found only seven studies [12–18] which met our inclusion criteria, of which three were peer reviewed [15, 17, 18]. DfE annual statistics related to educational outcomes in various formats have been published since 2000; [14] however, the six research studies were published very recently (2015, 2016 and 2018) [12, 13, 15–18]. All studies found that children in contact with CSC—as variously defined in each study—had worse educational outcomes than those without in concordance with the DfE statistical releases. However, all sources included in our review provided only a brief snapshot of educational outcomes of children in contact with CSC using blunt measures of this exposure.

A major limitation of the evidence base as to educational outcomes for children in contact with CSC is therefore its conspicuous absence. The studies included in this review all operationalise contact with CSC in crude ways that do not fully account for different longitudinal trajectories of contact with CSC (e.g., in need or not; or having had contact with CSC due to the child’s behaviour). Further, the measure of contact with CSC used by Henderson et al. [15] may have been subject to recall bias or desirability bias and all studies investigating attainment may be subject to some degree of selection bias arising from CLA and CiN being more likely to not sit their exams. Making better use of the longitudinal nature of administrative data can overcome these problems as data on all children and individuals can be linked over time to fully examine their ‘careers’ in the care system, as CiN and through school.

The primary focus of Sebba et al. [12], Luke and O’Higgins [17], O’Higgins [18] and Henderson et al. [15] is on GCSE results. Although GCSEs are undoubtedly important for children and young people, other educational outcomes are also relevant and should be examined. Attainment at younger and older ages are all important for a young person’s development, their career prospects and well-being into adulthood. Other educational outcomes that might mediate associations between CSC contact and attainment are also important and include absences and exclusions. Sebba et al. [12], Ellison and Hutchinson [16] and the DfE reports [13, 14] provide descriptive data on these measures and only limited conclusions can therefore be drawn about these aspects of children’s school careers: simple descriptions of outcomes in populations are insufficient to fully understand differences between groups and what might be causing them. Instead, multivariable methods that account for confounders, mediators and effect modifiers are needed.

This leads to another limitation of the studies: inadequate, or in the case of the DfE reports, no adjustment for confounding particularly regarding family background and socioeconomic position (SEP). Consideration of SEP, which is a complex, multifaceted construct not fully captured by a single indicator [21, 22], is especially important due to its strong relationship with CSC contact [23]. Sebba et al. [12] and Luke and O’Higgins [17] did adjust for free school meal eligibility and deprivation using the income domain affecting children index (IDACI) from the English indices of deprivation (see, e.g. [24] and DfE revised GCSE and equivalent results, characteristics national tables, Table CH1 [25]). Eligibility for free school meals is based on the parents’ receiving certain income-replacement benefits, and the IDACI is calculated with reference to the proportion of children in the child’s immediate neighbourhood who are in families that receive essentially the same benefits; the two are therefore measuring the same facet of SEP—income—and it is not clear whether inclusion of both in the model was appropriate. Furthermore, these measures, like each child’s trajectory through CSC services, are time-varying and Sebba et al.’s [12] analysis does not take this into account. As a technical point, Sebba et al. [12] treated the IDACI scores as a continuous measure in their descriptive analyses, which is arguably inappropriate as the scores are not interval scaled [26]. Further, the scores should not be used to examine how deprivation of areas has changed over time. Instead, the ranks should be used to examine changes in area deprivation relative to other areas [26]. Finally, in Sebba et al.’s modelling (Technical Appendix I, p 36 [12]), the score appears to be categorised as a binary variable but it is not clear on what basis this was done.

Henderson et al. [15] adjusted for parental occupational class and highest level of education, also in a non-time-varying fashion (though highest level of education is less likely to change). While taking into account different indicators of SEP is welcome, these studies do not directly capture all relevant SEP-related circumstances such as housing conditions, parental substance misuse or parental disability. Studies from Sweden have been better able to achieve this through data linkage to take into account, for example, birth-parental psychiatric illness and substance abuse in addition to parental education in studies examining long-term outcomes of children in contact with CSC [27–29]. While results from the Swedish studies cannot be generalised to the UK (in addition to the fact that they studied much older populations, law and practice are different in these jurisdictions), the methodology is instructive and attempts should be made to replicate it in the UK, which will require better quality data that enables linkage of children and their family members across health and CSC datasets [30].

### Strengths and limitations

As with all systematic reviews, there is a risk that we have under-identified relevant studies. However, such risk was mitigated by a very broad cross-disciplinary search strategy and by not limiting our search to peer-reviewed publications. We carried out extensive searches of reference lists and for grey literature. There might still be publication bias if any research was not published in either a peer-reviewed or non-peer-reviewed form but an overall deficit in the generation and use of research evidence regarding children who come into contact with CSC and the family justice system has been previously highlighted [30, 31]. Therefore although finding so few studies that met our eligibility criteria was surprising, it is unlikely that this is due to significant publication bias. Expanding our eligibility criteria to include studies from outside the UK is likely to have increased the range of literature we identified. However, variation in CSC and educational systems, social policies and the underlying populations make it difficult to combine studies from different countries. A limitation of our focus on UK-based studies is that our findings are not directly applicable to other countries. However, a corresponding strength is that this review includes the most relevant evidence base for policy related to improving educational outcomes for children in contact with CSC services in the UK.

### Policy implications

Based on the limited available evidence from official statistics and published literature, the vulnerable children served by CSC perform significantly worse than their peers. Potential causes for this are likely to be multifaceted, especially given that families in contact with CSC are heterogeneous in terms of their socioeconomic backgrounds and the causes for CSC involvement. Many children’s school achievement, for example, will be affected by pre-existing material deprivation or the experience of abuse or neglect [32]. In other cases, under-achievement may be attributable to the experience of CSC services themselves, particularly out-of-home care, for example where a child experiences multiple disruptive placements during his or her school career [32]. Whatever the cause, if children in contact with CSC services continue to be prevented from achieving their maximum potential, then the gap between these groups and the general population in terms of their careers, health and well-being throughout their entire life course will remain and could potentially widen.

The present systematic review throws into sharp relief the urgent need to conduct more population-level research into the educational prospects of children in contact with CSC services, especially from the perspective of the child’s journey through CSC services, which our review found was one of the weakest points in the existing evidence base. Given the difficulties associated with studying children and families in contact with CSC, administrative data will be particularly important. In fact, administrative data featured in most included studies in this review, and the authors are each engaged in on-going studies into educational outcomes of CLA and CiN using the National Pupil Database linked to CSC administrative data [33, 34]. Such studies will require appropriate control groups as well as more nuanced categorisation of contact with CSC that takes into account its various complexities. Paucity of research generation and use within the family legal system and CSC has been identified as a key barrier to improving children’s outcomes [30, 31]. Addressing the serious knowledge gap identified by this review will be the first step to helping some of our most vulnerable children and young people.

## Conclusion

Children in contact with CSC services on average perform significantly worse than their general population peers at school. However, despite employing a search across 16 databases supplemented with additional searches of other online sources, we found only seven studies that met our inclusion criteria. This review throws into sharp relief the urgent need to conduct more population-level research into the educational prospects of children in contact with CSC services.

## Additional files

Additional file 1: PRISMA checklist. (DOC 64 kb)

Additional file 2: Search strategy and results. (DOCX 35 kb)

Additional file 3: Update search strategy and results. (DOCX 49 kb)

Additional file 4: Routine Department for Education data collated. (XLSX 28 kb)

## Data Availability

Full search strings and results for each database are given in Additional files 2 and 3 and where numerical data were extracted (Table 3), these are in Additional file 4. No other datasets are available as no meta-analysis was conducted.

## Abbreviations

CiN: Child(ren) in need
CLA: Child(ren) looked after
CSC: Children’s Social Care
DfE: Department for Education
GCSE: General Certificate of Secondary Education
IDACI: Income Domain Affecting Children Index
UK: United Kingdom
