# Supplementary material for: Educational outcomes of children in contact with social care in England: a systematic review
Source: Syst Rev. 2019 Jun 28;8:155. doi: 10.1186/s13643-019-1071-z (PMC6599338; doi:10.1186/s13643-019-1071-z)
Supplement: Supplementary file 2 — Search strategy and results. (DOCX 35 kb) [file 13643_2019_1071_MOESM2_ESM.docx]

**ADDITIONAL FILE 2**

**Search strategy**

Database search conducted 21 December 2017.

***Ovid (Medline [and Epub Ahead of Print, In-Process & Other Non-Indexed Citations, Daily and Versions], Embase, PsycInfo and Social Policy & Practice together)***

|  | **String** | **Results** |
| --- | --- | --- |
| 1 | ("foster care" or "foster home" or "foster family" or "foster parent" or "foster carer" or "substitute family" or "family foster home" or "kinship care" or "child* in care" or "out#of#home care" or "looked#after" or "child* in need" or "vulnerable child*" or "social service*" or "Children Act 1989" or "Children (Northern Ireland) Order 1995" or "Children (Scotland) Act 1995").tw. | 70,111 |
| 2 | (educat* or school* or class* or college* or teach* or learn* or train* or diploma* or certificate* or tutor* or achiev* or perform* or academic).tw. | 13,925,436 |
| 3 | (England or English or UK or Britain or British or ALSPAC or BCS or LSYPE or MCS or NCDS or YCS).tw. | 1,045,231 |
| 4 | 1 and 2 and 3 | 3,210 |
| 5 | limit 4 to yr="1991 -Current" | 2,869 |
| 6 | limit 5 to english language [Limit not valid in Social Policy and Practice; records were retained] | 2,825 |
| 7 | limit 6 to (dissertation or journal article or letter or reviews) [Limit not valid in Embase, Ovid MEDLINE(R),Social Policy and Practice; records were retained] | 1,209 |

***Scopus***

| **String** | **Results** |
| --- | --- |
| TITLE-ABS(("foster care" or "foster home" or "foster family" or "foster parent" or "foster carer" or "substitute family" or "family foster home" or "kinship care" or "child* in care" or "out?of?home care" or "looked?after" or "child* in need" or "vulnerable child*" or "social service?" or "Children Act 1989" or "Children (Northern Ireland) Order 1995" or "Children (Scotland) Act 1995") AND (educat* or school* or class* or college* or teach* or learn* or train* or diploma* or certificate* or tutor* or achiev* or perform* or academic) AND (England or English or UK or Britain or British or ALSPAC or BCS or LSYPE or MCS or NCDS or YCS)) | 1,020 |
| AND ( LIMIT-TO ( PUBYEAR , 2018 ) OR LIMIT-TO ( PUBYEAR , 2017 ) OR LIMIT-TO ( PUBYEAR , 2016 ) OR LIMIT-TO ( PUBYEAR , 2015 ) OR LIMIT-TO ( PUBYEAR , 2014 ) OR LIMIT-TO ( PUBYEAR , 2013 ) OR LIMIT-TO ( PUBYEAR , 2012 ) OR LIMIT-TO ( PUBYEAR , 2011 ) OR LIMIT-TO ( PUBYEAR , 2010 ) OR LIMIT-TO ( PUBYEAR , 2009 ) OR LIMIT-TO ( PUBYEAR , 2008 ) OR LIMIT-TO ( PUBYEAR , 2007 ) OR LIMIT-TO ( PUBYEAR , 2006 ) OR LIMIT-TO ( PUBYEAR , 2005 ) OR LIMIT-TO ( PUBYEAR , 2004 ) OR LIMIT-TO ( PUBYEAR , 2003 ) OR LIMIT-TO ( PUBYEAR , 2002 ) OR LIMIT-TO ( PUBYEAR , 2001 ) OR LIMIT-TO ( PUBYEAR , 2000 ) OR LIMIT-TO ( PUBYEAR , 1999 ) OR LIMIT-TO ( PUBYEAR , 1998 ) OR LIMIT-TO ( PUBYEAR , 1997 ) OR LIMIT-TO ( PUBYEAR , 1996 ) OR LIMIT-TO ( PUBYEAR , 1995 ) OR LIMIT-TO ( PUBYEAR , 1994 ) OR LIMIT-TO ( PUBYEAR , 1993 ) OR LIMIT-TO ( PUBYEAR , 1992 ) OR LIMIT-TO ( PUBYEAR , 1991 ) ) | 901 |
| AND ( LIMIT-TO ( LANGUAGE , "English" ) ) | 887 |
| AND ( LIMIT-TO ( DOCTYPE , "ar" ) OR LIMIT-TO ( DOCTYPE , "re" ) OR LIMIT-TO ( DOCTYPE , "ip" ) ) | 811 |

Note that the number of results is cumulative: i.e., the entire search string concatenated together results in 811 hits.

***EBSCOhost (British Education Index, Education Abstracts, ERIC and Index to Legal Periodical and Books)***

|  | **String** | **Results** |
| --- | --- | --- |
| S1 | TI ( "foster care" or "foster home" or "foster family" or "foster parent" or "foster carer" or "substitute family" or "family foster home" or "kinship care" or "child* in care" or "out?of?home care" or "looked?after" or "child* in need" or "vulnerable child*" or "social service*" or "Children Act 1989" or "Children (Northern Ireland) Order 1995" or "Children (Scotland) Act 1995" ) OR AB ( "foster care" or "foster home" or "foster family" or "foster parent" or "foster carer" or "substitute family" or "family foster home" or "kinship care" or "child* in care" or "out?of?home care" or "looked?after" or "child* in need" or "vulnerable child*" or "social service*" or "Children Act 1989" or "Children (Northern Ireland) Order 1995" or "Children (Scotland) Act 1995" ) | 13,866 |
| S2 | TI ( educat* or school* or class* or college* or teach* or learn* or train* or diploma* or certificate* or tutor* or achiev* or perform* or academic ) OR ( educat* or school* or class* or college* or teach* or learn* or train* or diploma* or certificate* or tutor* or achiev* or perform* or academic ) | 3,182,935 |
| S3 | TI ( England or English or UK or Britain or British or ALSPAC or BCS or LSYPE or MCS or NCDS or YCS ) OR AB ( England or English or UK or Britain or British or ALSPAC or BCS or LSYPE or MCS or NCDS or YCS ) | 517,987 |
| S4 | S1 AND S2 AND S3 | 685 |
| S5 | Limit S4 to 1991 to present | 516 |
| S6 | Limit S5 to English | 219 |
|  | Exact duplicates automatically removed from results | 218 |

***ProQuest (Education Database, Social Science Database, ASSIA, International Bibliography of the Social Sciences, Sociology Database, Sociological Abstracts)***

| **String** | **Results** |
| --- | --- |
| (AB("foster care" or "foster home" or "foster family" or "foster parent" or "foster carer" or "substitute family" or "family foster home" or "kinship care" or "child* in care" or "out?of?home care" or "looked?after" or "child* in need" or "vulnerable child*" or "social service*" or "Children Act 1989" or "Children (Northern Ireland) Order 1995" or "Children (Scotland) Act 1995") OR TI("foster care" or "foster home" or "foster family" or "foster parent" or "foster carer" or "substitute family" or "family foster home" or "kinship care" or "child* in care" or "out?of?home care" or "looked?after" or "child* in need" or "vulnerable child*" or "social service*" or "Children Act 1989" or "Children (Northern Ireland) Order 1995" or "Children (Scotland) Act 1995" )) AND (AB(educat* or school* or class* or college* or teach* or learn* or train* or diploma* or certificate* or tutor* or achiev* or perform* or academic) OR TI(educat* or school* or class* or college* or teach* or learn* or train* or diploma* or certificate* or tutor* or achiev* or perform* or academic)) AND (AB(England or English or UK or Britain or British or ALSPAC or BCS or LSYPE or MCS or NCDS or YCS) OR TI(England or English or UK or Britain or British or ALSPAC or BCS or LSYPE or MCS or NCDS or YCS)) | 1,522 |
| Limit to 1991 to current | 1,374 |
| Limit to English | 1,318 |
| Limit to scholarly journals, trade journals, dissertations & theses or reports | 1,263 |
| Limit to article, feature, dissertation/thesis, report or review | 1,164 |
| Limit to UK regions | 157 |
| Duplicates automatically removed | 155 |

***Westlaw***

|  | **String** | **Results** |
| --- | --- | --- |
| 1 | "looked*after child!" OR "child! in care" OR "child! in need" OR "Children Act 1989" OR "Children Northern Ireland Order 1995" OR "Children Scotland Act 1995" | About 5,300* |
| 2 | educat! OR school! OR class! OR college! OR teach! OR learn! OR train! OR diploma! OR certificate! OR tutor! OR achiev! OR perform! OR academic | 1,273 |
| 3 | England or English or UK or Britain or British | 1,273 |
| 4 | empirical OR cohort OR case-control OR cross-sectional OR observational OR experiment OR "controlled trial" | 119 |
| 5 | Limit to 1991 to current | 112 |
| 6 | Limit to UK jurisdictions | 69 |
| 7 | Limit to article | 63 |
| 8 | Limit to following areas of law: children (under family and private life) OR Social services (under health and social security) OR Family proceedings (under legal system 🡪 civil procedure) | 30 |

Note that Westlaw’s search functionality is comparatively limited compared to the other databases, hence the altered search strategy. Strings 4 and 7 were inserted as Westlaw contains a large number of non-empirical studies as well as case law and case comments. String 1 was searched first. Subsequent strings were searched within results. * Exact number not given by Westlaw.

**Duplicate removal**

A total of 2,423 articles were identified. 2,393 of these were imported into Mendeley using RIS files (the 30 Westlaw articles could not be imported and were dealt with separately).

Mendeley automatically removed 466 definite duplicates on import, leaving 1,927 references. The de-duplication tool was then used to remove further duplicates. Mendeley found 608 potential duplicates across 276 sets. We checked these manually and merged where necessary.

Following deduplication, 1,593 references were retained. Including the 30 Westlaw results, there were therefore **1,623** **references** to screen.

**Snowball searches**

We also conducted forward and backward ‘snowball’ searches of the 13 publications eligible for full-text review. Google Scholar was used for the forward search (i.e. to identify papers citing the full-text review studies). This resulted in **one** additional study being included: Sebba et al. (2015) citing O’Sullivan (2007).

| **Full text**  **(First author, year)** | **Reason study was excluded** | **Sources in reference list**  **(backward search)** | **Sources cited by, as of 26^th^ April 2018**  **(forward search)** | **Number included** |
| --- | --- | --- | --- | --- |
| Colton (1995) | No gen pop comp | 16 | 29 | 0 |
| Downs (1997) | Not a primary study | 30 | 4 | 0 |
| Fernandez (2008) | Not UK | 32 | 104 | 0 |
| Goldson (1997) | Not a primary study | 51 | 4 | 0 |
| Guglani (2008) | Exposure not CSC | 34 | 11 | 0 |
| Hayden (1996) | Exposure not CSC | 27 | 21 | 0 |
| Heath (1994) | No gen pop comp | 42 | 157 | 0 |
| Henderson (2016) | INCLUDED | 68 | 7 | 0 |
| Montserrat (2017) | No gen pop comp | 30 | 2 | 0 |
| O’Sullivan (2007) | No gen pop comp | 33 | 52 | 1 |
| Oliver (2014) | Exposure not CSC | 34 | 10 | 0 |
| Stein (1994) | Not a primary study | 28 | 102 | 0 |
| West (2011) | Exposure not CSC | 36 | 32 | 0 |
|  | **Total** | **461** | **535** | **1** |

Gen pop comp: general population comparison group

CSC: children’s social care

See below for full references.

**Supplementary searches**

*Google Scholar*

An additional search of Google Scholar was also conducted on 26^th^ April 2018 with the following broad terms:

allintitle: ("looked-after children" OR "children in need") AND (education OR attainment OR school)

Limited to 1991 onwards, excluding patents

This resulted in 84 hits; two were potentially eligible but on full text screening were excluded.

*Websites*

The below websites were also searched for research reports. The search functions for these websites was limited and Boolean searches were not possible. Instead, we conducted separate searches for reports related to “looked-after children” and “children in need.” When a website did not have a search function, we hand-searched the research, publications and/or resources section(s) of the website.

| **Organisation** | **Website** | **Screened** | **Included** |
| --- | --- | --- | --- |
| Evidence for Policy and Practice Information and Co-ordinating Centre | <http://www.ucl.ac.uk/ioe/departments-centres/centres/evidence-for-policy-and-coordinating-centre> | 22 | 0 |
| Social Care Online | <https://www.scie-socialcareonline.org.uk/> | 63 | 1* |
| National Foundation for Educational Research | <https://www.nfer.ac.uk/> | 478 | 0 |
| The Fostering Network | <https://www.thefosteringnetwork.org.uk/> | 14 | 0 |
| CoramBAAF | <https://corambaaf.org.uk/> | 58 | 0 |
| The National Children's Bureau | <https://www.ncb.org.uk/> | 80 | 0 |
| NSPCC | <https://www.nspcc.org.uk/> | 561 | 0 |
| Grandparents Plus | <https://www.grandparentsplus.org.uk/> | 19 | 0 |
| Rees Centre | <http://reescentre.education.ox.ac.uk/> | 19 | 1* |
| Department for Education | <https://www.gov.uk/government/organisations/department-for-education> | 254 | 9 (2)† |
|  | **Total** | **1,568** | **2*** |

* Sebba et al. (2015) as also identified in the Google Scholar search. As this was a duplicate, it is not counted in the total.

† Eight were cross-sectional, annual releases which we aggregated as one study for this review. The Department for Education therefore contributed 2 studies.

**FULL REFERENCES FOR FULL TEXTS SCREENED**

Colton et al (1995) ‘Factors which influence the educational attainment of children in foster family care’ 7(1) *Community Alternatives: International Journal of Family Care* 15-36.

Downs et al (1997) ‘Linking health and education data to plan and evaluate services for children’ 102(7) *Arch Dis Child* 599-602.

Fernandez (2008) ‘Unravelling emotional, behavioural and educational outcomes in a longitudinal study of children in foster-care’ 38(7) *Br J Social Work* 1283-1301.

Goldson B (1997) ‘Locked out and locked up: state policy and the systemic exclusion of children “in need” in England and Wales’ 10(1) *Representing Children* 44-55.

Guglani et al (2008) ‘Mental health and educational difficulties in children in contact with children's social services’ 1 (2) *Child & Family Social Work* 188-196.

Hayden et al (1996) ‘Primary exclusions: Evidence for action’ 38(2) *Educational Research* 213-225.

Heath et al (1994) ‘Failure to escape: A longitudinal study of foster children's educational attainment’ 24(3) *Br J Social Work* 241-260.

Henderson et al (2016) ‘The Effects of Social Service Contact on Teenagers in England’ 26(4) *Research on Social Work Practice* 386-398.

Montserrat and Casas (2017) ‘The education of children and adolescents in out-of-home care: a problem or an opportunity? Results of a longitudinal study’ 21 *Eur J Social Work* 750-763.

O’Sullivan and Westerman (2007) ‘Closing the Gap: Investigating the Barriers to Educational Achievement for Looked after Children’ 31(1) *Adoption and Fostering* 13-20.

Also published as O’Sullivan and Westerman (2007) ‘Closing the Gap: Investigating the Barriers to Educational Achievement for Looked after Children’. In: Jackson (ed) *Pathways through education for young people in care: ideas from research and practice*. London: British Association for Adoption and Fostering, pp. 66-77.

Oliver et al (2014) ‘Configurations of early risk and their association with academic, cognitive, emotional and behavioural outcomes in middle childhood’ 49(5) *Social Psychiatry and Psychiatric Epidemiology* 723-732.

Stein (1994) ‘Leaving care, education and career trajectories’ 20(3) *Oxford Review of Education* 349-360.

West et al (2011) ‘Secondary school admissions in England 2001 to 2008: Changing legislation, policy and practice’ 37(1) *Oxford Review of Education* 1-20.
