# Supplementary material for: Educational outcomes of children in contact with social care in England: a systematic review
Source: Syst Rev. 2019 Jun 28;8:155. doi: 10.1186/s13643-019-1071-z (PMC6599338; doi:10.1186/s13643-019-1071-z)
Supplement: Supplementary file 3 — Update search strategy and results. (DOCX 49 kb) [file 13643_2019_1071_MOESM3_ESM.docx]

**ADDITIONAL FILE 3 – SEARCH UPDATE**

**Summary**

The original search strategy (see Additional File 2 for details) was updated on 7^th^ May 2019. A flow diagram for this updated search is given below. The exact search strings on the same databases were used. As before, we conducted foreword and backward snowball searches of full texts that were screened as well as a Google Scholar search and searches of relevant websites. Finally, we updated the forward snowball search that we originally conducted on the full texts we screened in the initial search. Details for all these searches are below. In sum, we screened an additional 1,211 records and included 3 new studies.

**Flow diagram**

Records identified through database search (7^th^ May 2019)
(n = 481)

Records after duplicates removed
(n = 272)

Records screened
(n = 272)

Records excluded
(n = 266)

Full-text articles assessed for eligibility
(n = 6)

Full-text articles excluded

(n = 3)

- Already identified by first search, n = 3

**Studies included in qualitative synthesis
(n = 3)**

Records identified through snowball search and screened (10^th^ May 2019)
(n = 136)

Records identified through supplementary searches and screened (10^th^ May 2019)
(n = 683 through websites; n = 8 through Google Scholar)

**Total records screened: 1,211**

**Total studies included: 3**

Full texts assessed for eligibility

(n = 0)

Full texts assessed for eligibility

(n = 0)

Full texts assessed for eligibility

(n = 0)

Record identified through updated snowball search of full texts screened in original search and screened

(n = 112)

**Search strategy**

Updated database search conducted 7 May 2019 for 2017 to current.

***Ovid (Medline [and Epub Ahead of Print, In-Process & Other Non-Index Citations, Daily and Versions], Embase, PsycInfo and Social Policy & Practice together)***

|  | **String** | **Results** |
| --- | --- | --- |
| 1 | ("foster care" or "foster home" or "foster family" or "foster parent" or "foster carer" or "substitute family" or "family foster home" or "kinship care" or "child* in care" or "out#of#home care" or "looked#after" or "child* in need" or "vulnerable child*" or "social service*" or "Children Act 1989" or "Children (Northern Ireland) Order 1995" or "Children (Scotland) Act 1995").tw. | 71,728 |
| 2 | (educat* or school* or class* or college* or teach* or learn* or train* or diploma* or certificate* or tutor* or achiev* or perform* or academic).tw. | 15,927,564 |
| 3 | (England or English or UK or Britain or British or ALSPAC or BCS or LSYPE or MCS or NCDS or YCS).tw. | 1,142,272 |
| 4 | 1 and 2 and 3 | 3,235 |
| 5 | limit 4 to yr="2017 -Current" | 272 |
| 6 | limit 5 to english language [Limit not valid in Social Policy and Practice; records were retained] | 260 |

***Scopus***

| **String** | **Results** |
| --- | --- |
| TITLE-ABS(("foster care" or "foster home" or "foster family" or "foster parent" or "foster carer" or "substitute family" or "family foster home" or "kinship care" or "child* in care" or "out?of?home care" or "looked?after" or "child* in need" or "vulnerable child*" or "social service?" or "Children Act 1989" or "Children (Northern Ireland) Order 1995" or "Children (Scotland) Act 1995") AND (educat* or school* or class* or college* or teach* or learn* or train* or diploma* or certificate* or tutor* or achiev* or perform* or academic) AND (England or English or UK or Britain or British or ALSPAC or BCS or LSYPE or MCS or NCDS or YCS)) | 1,160 |
| AND ( LIMIT-TO ( PUBYEAR , 2018 ) OR LIMIT-TO ( PUBYEAR , 2017 ) ) | 162 |
| AND ( LIMIT-TO ( LANGUAGE , "English" ) ) | 161 |
| AND ( LIMIT-TO ( DOCTYPE , "ar" ) OR LIMIT-TO ( DOCTYPE , "re" ) OR LIMIT-TO ( DOCTYPE , "ip" ) ) | 150 |

Note that the number of results is cumulative: i.e., the entire search string concatenated together results in 150 hits.

***EBSCOhost (British Education Index, Education Abstracts, ERIC and Index to Legal Periodical and Books)***

|  | **String** | **Results** |
| --- | --- | --- |
| S1 | TI ( "foster care" or "foster home" or "foster family" or "foster parent" or "foster carer" or "substitute family" or "family foster home" or "kinship care" or "child* in care" or "out?of?home care" or "looked?after" or "child* in need" or "vulnerable child*" or "social service*" or "Children Act 1989" or "Children (Northern Ireland) Order 1995" or "Children (Scotland) Act 1995" ) OR AB ( "foster care" or "foster home" or "foster family" or "foster parent" or "foster carer" or "substitute family" or "family foster home" or "kinship care" or "child* in care" or "out?of?home care" or "looked?after" or "child* in need" or "vulnerable child*" or "social service*" or "Children Act 1989" or "Children (Northern Ireland) Order 1995" or "Children (Scotland) Act 1995" ) | 14,847 |
| S2 | TI ( educat* or school* or class* or college* or teach* or learn* or train* or diploma* or certificate* or tutor* or achiev* or perform* or academic ) OR ( educat* or school* or class* or college* or teach* or learn* or train* or diploma* or certificate* or tutor* or achiev* or perform* or academic ) | 3,450,226 |
| S3 | TI ( England or English or UK or Britain or British or ALSPAC or BCS or LSYPE or MCS or NCDS or YCS ) OR AB ( England or English or UK or Britain or British or ALSPAC or BCS or LSYPE or MCS or NCDS or YCS ) | 549,641 |
| S4 | S1 AND S2 AND S3 | 874 |
| S5 | Limit S4 to 2017 to present | 93 |
| S6 | Limit S5 to English | 48 |
|  | Exact duplicates automatically removed from results | 42 |

***ProQuest (Education Database, Social Science Database, ASSIA, International Bibliography of the Social Sciences, Sociology Database, Sociological Abstracts)***

| **String** | **Results** |
| --- | --- |
| (AB("foster care" or "foster home" or "foster family" or "foster parent" or "foster carer" or "substitute family" or "family foster home" or "kinship care" or "child* in care" or "out?of?home care" or "looked?after" or "child* in need" or "vulnerable child*" or "social service*" or "Children Act 1989" or "Children (Northern Ireland) Order 1995" or "Children (Scotland) Act 1995") OR TI("foster care" or "foster home" or "foster family" or "foster parent" or "foster carer" or "substitute family" or "family foster home" or "kinship care" or "child* in care" or "out?of?home care" or "looked?after" or "child* in need" or "vulnerable child*" or "social service*" or "Children Act 1989" or "Children (Northern Ireland) Order 1995" or "Children (Scotland) Act 1995" )) AND (AB(educat* or school* or class* or college* or teach* or learn* or train* or diploma* or certificate* or tutor* or achiev* or perform* or academic) OR TI(educat* or school* or class* or college* or teach* or learn* or train* or diploma* or certificate* or tutor* or achiev* or perform* or academic)) AND (AB(England or English or UK or Britain or British or ALSPAC or BCS or LSYPE or MCS or NCDS or YCS) OR TI(England or English or UK or Britain or British or ALSPAC or BCS or LSYPE or MCS or NCDS or YCS)) | 1,580 |
| Limit to 2017 to current | 78 |
| Limit to English | 77 |
| Limit to scholarly journals, trade journals, reports or other sources | 74 |
| Limit to article, feature or review | 65 |
| Limit to UK regions | 24 |

***Westlaw***

|  | **String** | **Results** |
| --- | --- | --- |
| 1 | "looked*after child!" OR "child! in care" OR "child! in need" OR "Children Act 1989" OR "Children Northern Ireland Order 1995" OR "Children Scotland Act 1995" | About 6,100* |
| 2 | educat! OR school! OR class! OR college! OR teach! OR learn! OR train! OR diploma! OR certificate! OR tutor! OR achiev! OR perform! OR academic | 1,476 |
| 3 | England or English or UK or Britain or British | 1,476 |
| 4 | empirical OR cohort OR case-control OR cross-sectional OR observational OR experiment OR "controlled trial" | 135 |
| 5 | Limit to 2017 to current | 15 |
| 6 | Limit to UK jurisdictions | 12 |
| 7 | Limit to article | 12 |
| 8 | Limit to following areas of law: children (under family and private life) [No other relevant areas of law] | 5 |

Note that Westlaw’s search functionality is comparatively limited compared to the other databases, hence the altered search strategy. Strings 4 and 7 were inserted as Westlaw contains a large number of non-empirical studies as well as case law and case comments. String 1 was searched first. Subsequent strings were searched within results. * Exact number not given by Westlaw.

**Duplicate removal**

A total of 481 articles were identified. 476 of these were imported into Mendeley using RIS files (the 5 Westlaw articles could not be imported and were dealt with separately).

Mendeley automatically removed 105 definite duplicates on import, leaving 321 references. The de-duplication tool was then used to remove further duplicates (89 duplicates across 42 sets). We checked these manually and after de-duplication there were 324 records. We removed a further 55 records whose date of entry into Mendeley indicated they were records which had already been screened as part of the first search. Finally, we merged four more records (across two sets) that we discovered during screening but which the deduplication tool did not detect. This left 267 documents. Including the 5 Westlaw results, there were therefore **272 additional references to screen**.

**New studies included**

We screened 6 studies and included 3 (the other 3 were already identified in the original search).

**Snowball searches**

We also conducted forward and backward ‘snowball’ searches of 3 of the 6 publications eligible for full-text review. (The other 3 were already identified in the first search and therefore already screened.) Google Scholar was used for the forward search (i.e. to identify papers citing the full-text review studies).

| **Full text**  **(First author, year)** | **Reason study was excluded** | **Sources in reference list**  **(backward search)** | **Sources cited by, as of 10^th^ May 2019**  **(forward search)** | **Number included** |
| --- | --- | --- | --- | --- |
| DfE, 2017 | Already in first search | N/A | N/A | N/A |
| DfE, 2018 | Already in first search | N/A | N/A | N/A |
| Montserrat, 2018 | Already in first search | N/A | N/A | N/A |
| Ellison, 2018 | Included | 10 | 0 | 0 |
| Luke, 2018 | Included | 71 | 2 | 0 |
| O’Higgins, 2019 | Included | 55 | 0 | 0 |
|  | **Total** | **136** | **2** | **0** |

Gen pop comp: general population comparison group

CSC: children’s social care; DfE Department for Education

See below for full references.

**Supplementary searches**

*Google Scholar*

An additional search of Google Scholar was also conducted on 10 May 2019 with the following broad terms:

allintitle: ("looked-after children" OR "children in need") AND (education OR attainment OR school)

Limited to 2017 onwards, excluding patents

This resulted in 8 hits, none of which were potentially eligible on abstract screening.

*Websites*

The below websites were also searched for research reports published since the last search. The search functions for these websites was limited and Boolean searches were not possible. Instead, we conducted separate searches for reports related to “looked-after children” and “children in need.” When a website did not have a search function, we hand-searched the research, publications and/or resources section(s) of the website.

| Organisation | **Website** | **Screened** | **Included** |
| --- | --- | --- | --- |
| Evidence for Policy and Practice Information and Co-ordinating Centre | <http://www.ucl.ac.uk/ioe/departments-centres/centres/evidence-for-policy-and-coordinating-centre> | 10 | 0 |
| Social Care Online | <https://www.scie-socialcareonline.org.uk/> | 104 | 0* |
| National Foundation for Educational Research | <https://www.nfer.ac.uk/> | 37 | 0 |
| The Fostering Network | <https://www.thefosteringnetwork.org.uk/> | 2 | 0 |
| CoramBAAF | <https://corambaaf.org.uk/> | 99 | 0 |
| The National Children's Bureau | <https://www.ncb.org.uk/> | 6 | 0* |
| NSPCC | <https://www.nspcc.org.uk/> | 120 | 0 |
| Grandparents Plus | <https://www.grandparentsplus.org.uk/> | 150 | 0 |
| Rees Centre | <http://reescentre.education.ox.ac.uk/> | 5 | 0 |
| Department for Education | <https://www.gov.uk/government/organisations/department-for-education> | 150 | 0* |
|  | **Total** | **683** | **0** |

* The Department for Education routine statistical releases, which were included in the original search, were found on Social Care Online and the Department for Education website; they have been excluded from these figures. The report by Ellison and Hutchinson (2018) was identified on the National Children’s Bureau website but was also excluded from these figures as it was already identified in the main update search.

**Updated snowball search for the full-texts screened in the initial search**

Using Google Scholar, restricting search to 2017 to present.

| **Full text**  **(First author, year)** | **Sources cited by, as of 10^th^ May 2019**  **(forward search for 2017 to current)** | **Number included** |
| --- | --- | --- |
| Colton (1995) | 2 | 0 |
| Downs (1997) | 45 | 0 |
| Fernandez (2008) | 17 | 0 |
| Goldson (1997) | 0 | 0 |
| Guglani (2008) | 1 | 0 |
| Hayden (1996) | 0 | 0 |
| Heath (1994) | 13 | 0* |
| Henderson (2016) | 0 | 0 |
| Montserrat (2017) | 6 | 0 |
| O’Sullivan (2007) | 8 | 0 |
| Oliver (2014) | 8 | 0 |
| Stein (1994) | 6 | 0 |
| West (2011) | 6 | 0 |
| **Total** | **112** | **0** |

See additional file 2 for full references.

* Luke and O’Higgins (2018) identified as citing Heath (1994) but not included in these numbers as it was already identified in the main search.

**FULL REFERENCES FOR FULL TEXTS SCREENED**

Department for Education (2017) Outcomes for children looked after by local authorities in England, 31 March 2016. <https://www.gov.uk/government/statistics/outcomes-for-children-looked-after-by-las-31-march-2016> (accessed 10 May 2019).

Department for Education (2018) Outcomes for children looked after by local authorities in England, 31 March 2017. <https://www.gov.uk/government/statistics/outcomes-for-children-looked-after-by-las-31-march-2017> (accessed 10 May 2019).

Ellison and Hutchinson (2018) Children missing education: children missing from education in England 2016-17. <https://www.ncb.org.uk/resources-publications/resources/children-missing-education> (accessed 10 May 2019).

Luke and O’Higgins (2018). ‘Is the Care System to Blame for the Poor Educational Outcomes of Children Looked After? Evidence from a Systematic Review and National Database Analysis’ 43(2) *Children Australia* 135-151.

Montserrat and Casas (2017) ‘The education of children and adolescents in out-of-home care: a problem or an opportunity? Results of a longitudinal study’ 21 *Eur J Social Work* 750-763.

O’Higgins (2019) ‘Analysis of care and education pathways of refugee and asylum-seeking children in care in England: Implications for social work’ 28(1) *Int J Social Work* 53-62.
